# Reactivity and Passivation of Fe Nanoclusters on *h‐*BN/Rh(111)

**DOI:** 10.1002/chem.202102590

**Published:** 2021-09-02

**Authors:** Natalie J. Waleska, Fabian Düll, Philipp Bachmann, Felix Hemauer, Johann Steinhauer, Christian Papp

**Affiliations:** ^1^ Lehrstuhl für Physikalische Chemie II Universität Erlangen-Nürnberg Egerlandstr. 3 91058 Erlangen Germany

**Keywords:** heterogeneous catalysis, iron, model catalysis, nanocluster array, XPS

## Abstract

The reactivity of iron nanocluster arrays on *h‐*BN/Rh(111) was studied using in situ high‐resolution X‐ray photoelectron spectroscopy. The morphology and reactivity of the iron nanoclusters (Fe‐NCs) were investigated by CO adsorption. On‐top and hollow/edge sites were determined to be the available adsorption sites on the as‐prepared Fe‐NCs and CO dissociation was observed at 300 K. C‐ and O‐precovered Fe‐NCs showed no catalytic activity towards CO dissociation because the hollow/edge sites were blocked by the C and O atoms. Therefore, these adsorption sites were identified to be the most active sites of the Fe‐NCs.

## Graphical abstract

**The reactivity of iron nanoclusters** (Fe‐NCs) on *h*‐BN/Rh(111) was investigated using in situ high‐resolution X‐ray photoelectron spectroscopy and CO as a probe molecule. The hollow/edge sites were determined to be the most active sites of the clusters. Dissociation of CO was only observed on the as‐prepared Fe‐NCs, while no catalytic activity towards CO dissociation was found for the precovered clusters as a result of blocking of the most active sites by adatoms.

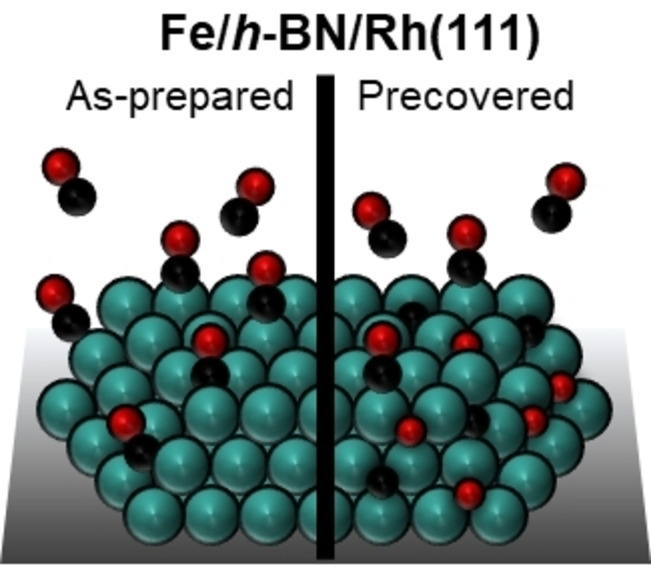

## Introduction

Around the 1980s, numerous studies were performed on the catalytic activity of iron catalysts. Especially, in regard to the Fischer‐Tropsch synthesis, the adsorption of CO and H_2_ on the low‐index iron single‐crystal surfaces Fe(100),[^1^, ^2^, ^3^, ^4^] Fe(110)[^5^, ^6^, ^7^, ^8^] and Fe(111)[^9^, ^10^] was investigated to gain a better understanding in the mechanism of the catalytic reaction. It was found that these iron surfaces are highly reactive towards CO dissociation at room temperature.[^1^, ^6^, ^7^, ^8^] Adsorption of molecular CO was only possible below 300 K, while dissociative adsorption was observed at higher temperatures.[^1^, ^8^, ^9^] Due to the high reactivity of iron catalysts, impurities like carbon and oxygen are commonly observed on iron surfaces.[^3^, ^6^] The catalytic activity of iron catalysts is strongly affected by such modifiers: Carbon, oxygen and sulfur inhibit dissociation of CO, while potassium adatoms were found to enhance CO dissociation.[^1^, ^2^, ^5^] Besides its high catalytic activity, iron is nontoxic and – as an abundant element – cheap in comparison to other transition metals.[^7^, ^11^, ^12^, ^13^]

Iron clusters are particularly interesting as they can be used in electrochemical devices,[^11^, ^14^] or for high density data storage applications due to their magnetic properties and the formation of the high magnetic L1_0_ phase in alloy systems with platinum.[^15^, ^16^] Moreover, nanoclusters are in general highly reactive catalysts as they additionally possess a variety of adsorption sites like corner and edge sites, which are not available on perfect single‐crystal surfaces.^[17]^ The strong influence of these structural differences on the catalytic activity in comparison to bulk surfaces was shown by Lei et al.^[18]^ for the epoxidation of propylene using silver catalysts. While unpromoted Ag_3_ clusters and Ag nanoparticles (∼3.5 nm) showed high catalytic activity even at low temperature, this is different for the utilization of bulk silver surfaces.^[18]^ Also, iron clusters were recently investigated experimentally[^12^, ^19^] and theoretically.[^14^, ^20^] In a noncontact atomic force microscopy study, Berwanger et al.^[12]^ investigated the interaction of iron clusters and CO using CO‐terminated tips. This experiment showed that the interaction between the CO tip and the particular iron atom increases with decreasing number of adjacent Fe atoms.^[12]^ From this, the corner and edge sites of the iron clusters were determined to be the most active ones.^[12]^

One successful route to efficiently study the properties of metal clusters is to prepare ordered nanocluster arrays on corrugated templates such as the 2D materials graphene or hexagonal boron nitride (*h‐*BN) on certain transition metal surfaces. Due to the lattice mismatch, lateral differences in the strength of interaction between the 2D material and the metal surface occur, leading to a regularly corrugated Moiré‐patterned nanosheet.[^21^, ^22^, ^23^, ^24^] Metal nanocluster arrays deposited onto such ordered structured substrates have a narrow size distribution and thus serve as model catalysts, representing an innovative approach to overcome the material gap to commercial catalysts.[^25^, ^26^] Various metal nanoclusters were already investigated on graphene and *h‐*BN, for instance Pd and Pt on graphene/Rh(111)[^17^, ^21^] and *h‐*BN/Rh(111),^[27]^ Au on *h‐*BN/Rh(111),[^28^, ^29^] and Ni and Fe on graphene/Rh(111).^[30]^ In addition, computational studies addressing the mobility of metal clusters on *h‐*BN were performed.^[31]^

In this work, iron nanocluster arrays (Fe‐NCs) on *h‐*BN/Rh(111) were studied by using CO as a probe molecule. The reaction of CO on the Fe‐NCs was monitored by in situ high‐resolution X‐ray photoelectron spectroscopy (HR‐XPS). Besides the investigation of CO adsorption on as‐prepared Fe‐NCs, also clusters precovered with C and O were studied, in order to determine the influence of adatoms on the catalytic behavior. Temperature programmed XPS was used to study the thermal stability and structural changes of the Fe‐NCs.

## Experimental Section

The experiments were performed at the synchrotron facility BESSY II of the Helmholtz‐Zentrum Berlin at the UE56/2‐PGM‐2 beamline. We used our own transportable UHV apparatus, which consists of an analysis chamber and a preparation chamber. A hemispherical photoelectron energy analyzer and a three‐stage supersonic molecular beam are connected to the analysis chamber. CO was dosed by the molecular beam. The preparation chamber is equipped with electron beam evaporators, which are used to evaporate Fe. The deposition rate during evaporation was calibrated by a quartz crystal microbalance. Furthermore, a sputter gun for sample cleaning is attached to the preparation chamber.

A sample temperature range of 140–1400 K is achieved by resistive heating and liquid nitrogen cooling. Additionally, a tungsten filament is mounted on the back site of the sample, which allows for heating of the sample up to 550 K, while minimizing the magnetic field observed for resistive heating. A heating ramp of 0.5 K/s was used to perform temperature programmed XPS (TPXPS).

The *h‐*BN layer was prepared by chemical vapor deposition of borazine at a pressure of 2×10^−8^ mbar at 1050 K. The growth and uniformity of the *h‐*BN layer was checked by XPS. 1.4 ML Fe were deposited at 150 K by electron beam evaporation and the deposition rate was calibrated with the QCM. In general, 1 ML of Fe would correspond to 144 atoms per Moiré unit cell according to the lattice mismatch of *h*‐BN on Rh(111) of (13×13)/(12×12).[^23^, ^27^] As the amount of empty pores in the N 1s core level region is 55±5 % (see Supporting Information Figure S5), the number of atoms per cluster, and thus also per Moiré cell, is ∼370 Fe atoms. As the average cluster height for a coverage of 1 ML of deposited metal is 4–5 layer,^[32]^ we expect a cluster height of ∼1 nm and a cluster diameter of ∼3 nm. To exclude the presence of larger Fe clusters, the sample was analyzed microscopically by SEM (see Supporting Information Figure S6, S7 and S8). To convert the intensity of the C 1s spectra into ML, the spectra were integrated. These integrals were compared to the integrals of reference spectra of a saturated CO layer on Pt(111), which corresponds to a coverage of 0.5 ML as it reveals a c(4x2) structure at saturation.^[33]^

The XP spectra were recorded at normal emission and the binding energies are referenced to the Fermi energy. The resolution of the C 1s and O 1s spectra is 180 and 300 meV, at excitation energies of 380 and 650 eV, respectively. For quantitative analysis, the spectra were fitted with a set of asymmetric Doniach‐Ŝunjić functions convoluted with Gaussian functions after a linear background was subtracted. The fit parameters are listed in the Supporting Information.

## Results and Discussion

### CO adsorption and TPXPS on as‐prepared Fe nanoclusters

**Figure 1:**
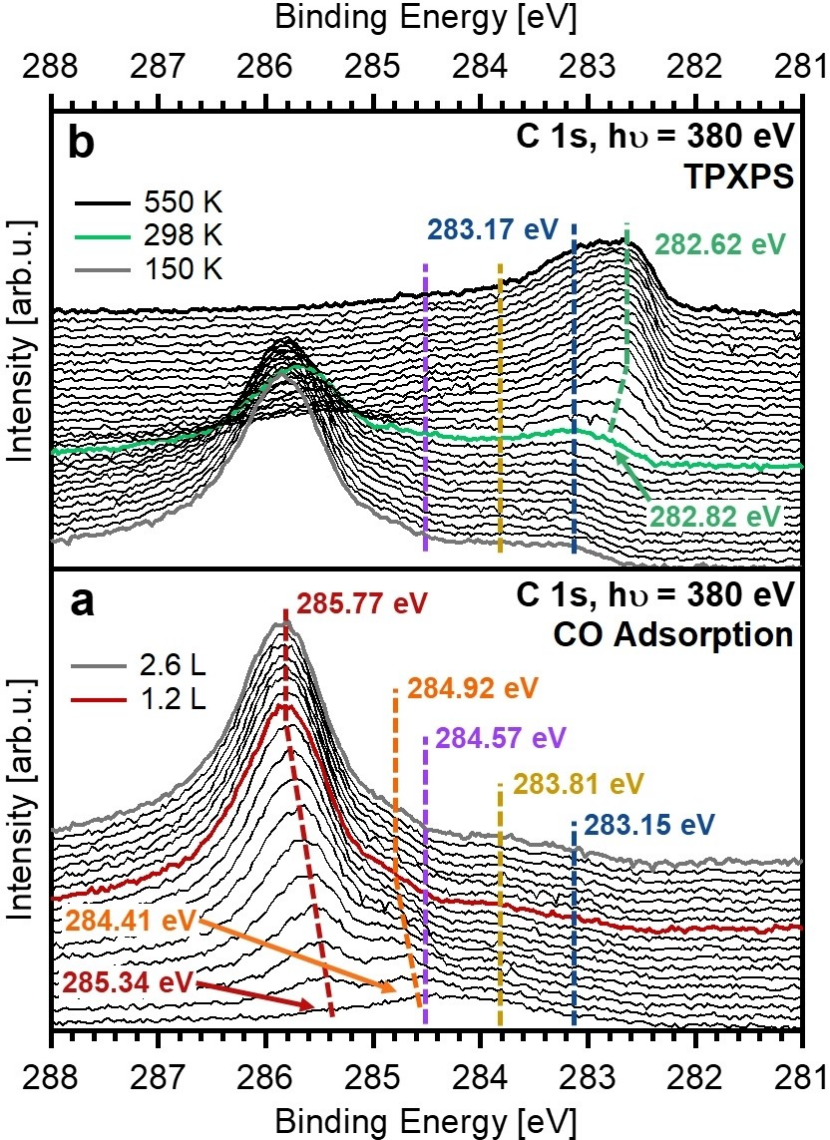
(a) C 1s spectra collected during CO adsorption on the as‐prepared Fe nanoclusters. The dashed lines indicate the shift of the CO^top^ (red) and CO^hollow/edge^ (orange) species during adsorption. Minor amounts of C^Gr^ and C^Rh^ are shown by the purple and ocher dashed lines, respectively. CO saturation was reached at 1.2 L and CO exposure was stopped at 2.6 L. (b) C 1s spectra collected during TPXPS of the as‐prepared Fe nanoclusters. The dashed lines show the Fe_3_C (blue) and Fe_3_C^surface^ (green) species.

**Figure 2:**
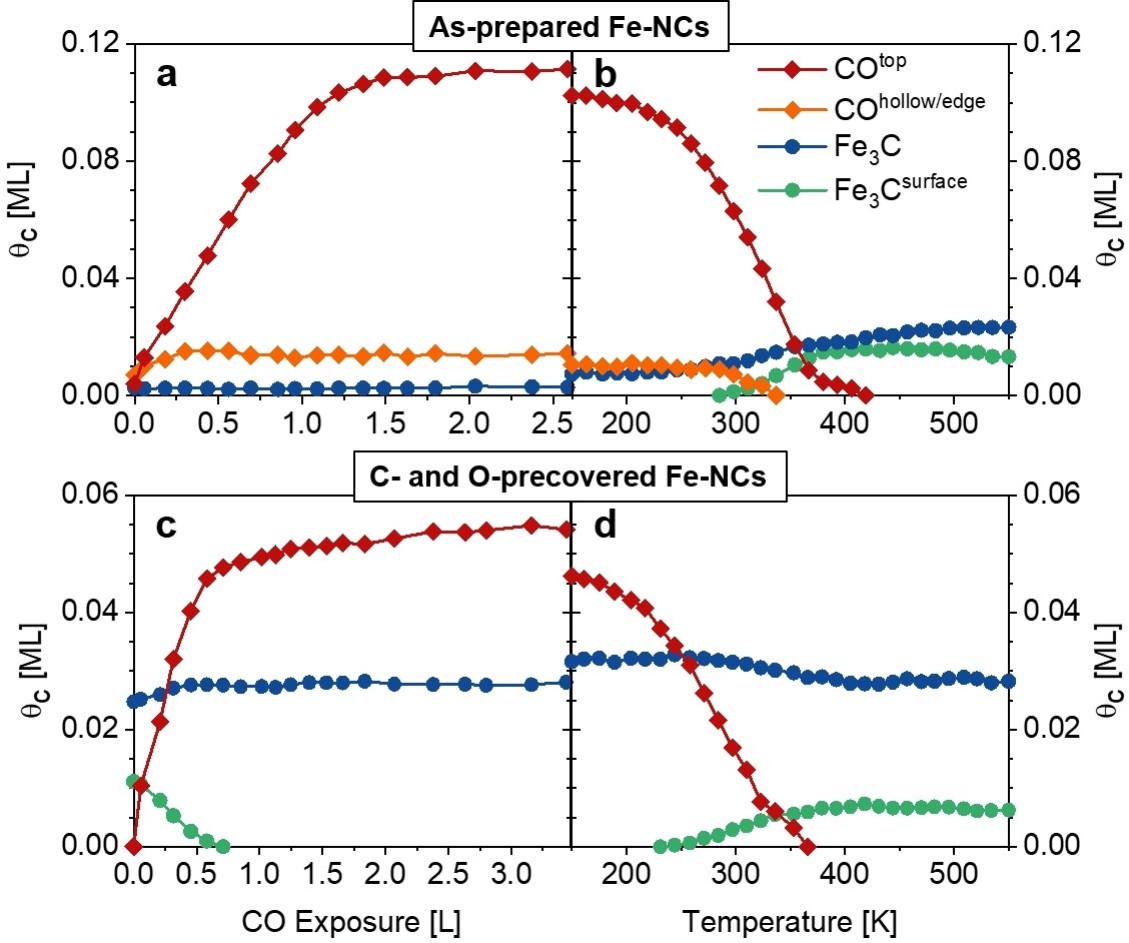
Quantitative analysis of the C 1s spectra during (a) CO adsorption on as‐prepared Fe nanoclusters (θ_C, max_=0.14 ML) and (b) TPXPS (θ_C, max_=0.13 ML), (c) CO adsorption on C‐ and O‐precovered Fe nanoclusters (θ_C, max_=0.09 ML) and (d) TPXPS (θ_C, max_=0.09 ML). The difference in coverage in between the adsorption and TPXPS is due to beam‐induced dissociation. For clarity, C^Gr^ and substrate carbide (C^Rh^) are not shown in these figures.

**Figure 3:**
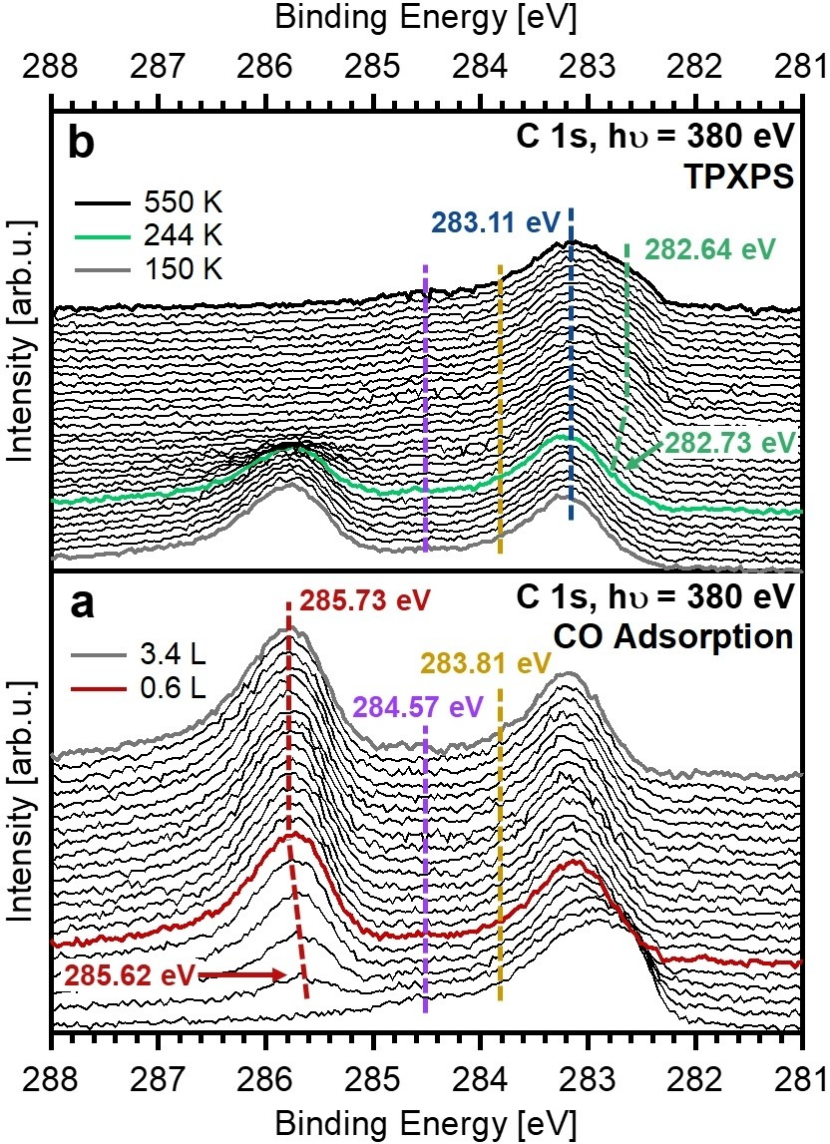
(a) C 1s spectra collected during CO adsorption on the C‐ and O‐precovered Fe nanoclusters. The dashed lines indicate the shift of the CO^top^ (red) species during adsorption. Minor amounts of C^Gr^ and C^Rh^ are shown by the purple and ocher dashed lines, respectively. CO saturation was reached at 0.6 L and CO exposure was stopped at 3.4 L. (b) C 1s spectra collected during TPXPS of the C‐ and O‐precovered Fe nanoclusters. The dashed lines show the Fe_3_C (blue) and Fe_3_C^surface^ (green) species.

The as‐prepared Fe‐NCs were exposed to the CO beam at 150 K. Please note that small amounts of carbon (0.01 ML) and oxygen (0.05 ML) were already present on the sample at the beginning of the experiment (Figure 1, 2 and Figure S1 in Supporting Information). In Figure 1a, the spectra recorded during CO adsorption are shown. With increasing exposure, we find a main signal shifting from 285.34 to 285.77 eV, and a shoulder at lower binding energies, shifting from 284.41 to 284.92 eV. The main signal at 285.77 eV (red) is assigned to CO adsorbed at on‐top sites, while the shoulder at 284.92 eV (orange) results from the adsorption of CO at the hollow sites, as well as the edge sites of the clusters. The assignment of the CO C 1s signals was made in comparison to the most favorable adsorption sites reported for the most stable low‐index iron surfaces (Table 1). These are the four‐fold hollow sites on Fe(100)[^1^, ^2^, ^34^] and the on‐top sites on Fe(110);[^7^, ^35^] this assignment is supported by quantum chemical calculations.[^36^, ^37^] The energetic order of the CO C 1s peaks is in line with observations studied for other transition metal surfaces, like Pt(111),^[38]^ Rh(111)^[39]^ and Ni(111),^[40]^ with the C 1s binding energy of CO adsorbed at on‐top sites higher than that of bridge and hollow species. Also, for step and edge adsorption sites on metal nanoclusters lower C 1s binding energies are found than for on‐top sites.[^17^, ^41^, ^42^] In our experiment, it was not possible to distinguish between CO adsorption at hollow sites or edge sites on the iron clusters due to their very similar binding energy. The binding energy difference between the CO^top^ and CO^hollow/edge^ species remains constant 0.90±0.05 eV during the shift of these signals. This shift is assigned to increasing lateral interactions of the adsorbed CO molecules at increasing coverage. In addition, two small features are observed at 284.57 and 283.81 eV (Figure 1 and 3), which are assigned to minor amounts of graphitic carbon (C^Gr^, see below) and a carbide species (C^Rh^), respectively. The latter is assigned to carbidic carbon on Rh(111), as a result of *h‐*BN preparation; it does not show any changes throughout the experiments. A similar species was also observed by Düll et al.^[42]^ after the preparation of graphene.

**Table 1:** Binding energies of CO adsorbed on as‐prepared Fe nanoclusters on *h‐*BN/Rh(111) and low‐index single‐crystal surfaces Fe(100) and Fe(110) given in eV. Binding energies of this work are referenced to the Fermi level.

	Fe/ *h‐*BN/Rh(111)	Fe(100)			Fe(110)	
References	*This work*	^[3]^	^[2]^	^[1]^	^[5]^	^[6]^
CO^top^	285.77	–	–	–	285.8	285.9
CO^hollow/edge^	284.92	–	284.1	284.8	–	–
C^Gr^	284.57	284.6			–	285.0
C^Rh^	283.81	–			–	–
Fe_3_C	283.12	283.5^[b]^ 283.1^[c]^			–	283.3
Fe_3_C^surface^	282.62	282.6	282.0^[a]^	282.3^[a]^	–	–

[a] dissociated CO; [b] iron‐chromium carbide; [c] iron‐manganese carbide.

The quantitative analysis of the spectra in Figure 2a shows that the CO^hollow/edge^ sites (orange diamonds) saturate first with a maximum of 0.015 ML at an exposure of 0.5 L. CO^top^ (red diamonds) increases until saturation at ∼1.2 L and reaches a maximum coverage of 0.11 ML, while the CO^hollow/edge^ coverage slightly decreases to 0.013 ML. Notably, there is already a small Fe_3_C coverage of 0.003 ML that does not change during adsorption (see below). The minor signals of C^Gr^ (0.003 ML) and C^Rh^ (0.007 ML) are not included in Figure 2.

After CO adsorption, temperature programmed XPS was performed. Figure 1b shows the C 1s spectra recorded upon annealing to 550 K. With increasing temperature, the C 1s signal of CO decreases due to partial desorption. At 298 K, two new signals emerge at 283.17 (blue) and 282.82 eV (green), which bare attribute to CO dissociation and are assigned to iron carbide, Fe_3_C. While the high binding energy species stems from the bulk atoms, the lower binding energy species is assigned to surface atoms, which display a surface core level shift (SCLS); such shifts are commonly found for transition metal surfaces, e. g. rhodium and palladium,^[43]^ as well as transition metal carbides.^[44]^ Also, for Pt nanoclusters on *h*‐BN/Rh(111) with a coverage as low as 0.019 ML the surface‐ and bulk‐like species can be identified clearly, corresponding to the top and lower layers of the clusters.^[27]^ Fe_3_C^surface^ shifts to lower binding energies with increasing temperature. This is assumed to be either a temperature dependent shift or a result of the formation of more Fe_3_C.

The quantitative analysis of the TPXPS is shown in Figure 2b. Upon heating the CO^top^ species starts to decrease at ∼200 K as a result of CO desorption and dissociation and vanishes at 400 K; the CO^hollow/edge^ species initially remains nearly constant and also starts to decrease at ∼290 K due to further desorption and dissociation. At the same time, both Fe_3_C species start to increase. CO dissociation is supported by the spectra in the O 1s core level region (Supporting Information Figure S1), where the clear signature of the transition of molecular CO at 531.8 eV to atomic oxygen at 529.8 eV is observed. The desorption temperature of CO was estimated to be 313 K (at 50 % of maximum CO coverage, see Figure S2). At temperatures above 400 K, the Fe_3_C^surface^ decreases from 0.016 to 0.013 ML, while Fe_3_C further increases up to 0.023 ML. This is attributed to a restructuring of the Fe_3_C containing NCs. Notably, the differences in coverage between the CO adsorption (last data point in Figure 2a) and temperature‐programmed experiment (first data point in Figure 2b) are assigned to beam‐induced dissociation.

### CO adsorption and TPXPS on C‐ and O‐precovered Fe nanoclusters

In a next step, the dissociation products formed upon heating of the CO‐covered Fe‐NCs to 550 K (previous section) were used as starting point to study the influence of C and O adatoms on the adsorption of CO and the catalytic activity of the Fe‐NCs. Figure 3a shows the corresponding C 1s spectra during adsorption of CO on the C‐ and O‐precovered clusters, and Figure 3b the subsequent TPXPS experiment. From the data in Figure 3a and the quantitative analysis in Figure 2c, it is evident that CO merely adsorbs at on‐top sites (285.73 eV) and starts to saturate at ∼0.6 L. Again, the C 1s peak of CO^top^ shifts to higher binding energy with increasing exposure. The maximum coverage of CO^top^ is 0.054 ML that is only ∼50 % of that observed on the as‐prepared Fe‐NCs. Since no adsorption was found at hollow/edge sites on the C‐ and O‐precovered Fe‐NCs, we conclude that these sites must be blocked by the carbon and oxygen adatoms. The presence of carbon and oxygen atoms also hinders the adsorption of CO at on‐top sites, resulting in the significantly smaller CO^top^ coverage. This is in agreement with the works of Cameron and Dwyer^[45]^ who reported a smaller amount of CO being readsorbed after CO dissociation on the Fe(100) surface and Fu et al.^[46]^ who found a decrease in CO adsorption on FeO islands on Pt(111) with increasing coverage of the FeO islands.

During CO adsorption on the precovered Fe‐NCs, the Fe_3_C^surface^ signal vanishes as the surface becomes increasingly covered with CO (Figure 2c, green) as is typically observed for surface core level shifted peaks.^[44]^ We do not find a new contribution due to the formation of a newly formed bond, suggesting that the binding energy now is similar to the Fe_3_C (blue). Indeed, the Fe_3_C signal increases slightly from 0.025 to 0.028 ML. An evaluation of the damping of the iron carbide signals by the adsorbed CO shows that the amount of Fe_3_C stays constant during CO adsorption (Figure S3).

The subsequent TPXPS spectra are shown in Figure 3b, the corresponding quantitative analysis in Figure 2d. Upon heating, the CO^top^ species starts to decrease at ∼200 K as a result of CO desorption, with no sign of CO dissociation, and vanishes at 360 K. The signal of the Fe_3_C^surface^ species reemerges at ∼244 K and shifts again to lower binding energies by 0.09 eV. This shift of the C 1s peak of Fe_3_C^surface^ is smaller than the shift of 0.2 eV for the as‐prepared Fe‐NCs. It is assumed that the Fe_3_C^surface^ signal shifts less because the amount of carbide did not change during the TPXPS for the latter experiment, in contrast to the as‐prepared Fe‐NCs, where Fe_3_C was formed during the TPXPS by CO dissociation. One other factor could be the smaller amount of CO adsorbed on the precovered clusters in comparison to the as‐prepared Fe‐NCs, which is also reflected in a smaller shift of the CO^top^ signal. With the desorption of CO and the reemergence of the surface carbide, the Fe_3_C signal decreases again from 0.032 to 0.028 ML.

**Figure 4:**
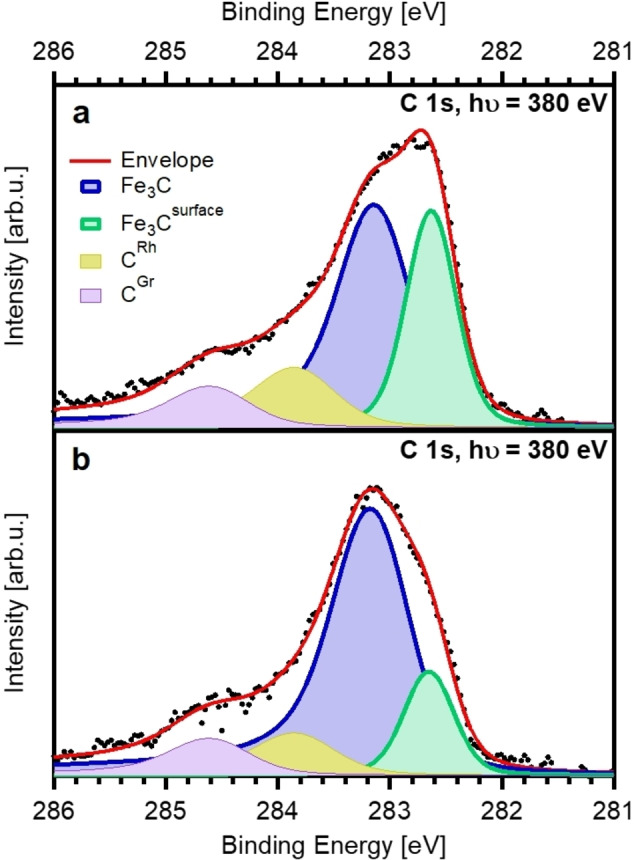
XP spectra of (a) as‐prepared and (b) precovered Fe‐NCs recorded at 550 K during TPXPS. Included are the fits of the Fe_3_C (blue) and Fe_3_C^surface^ (green) peak, as well as the C^Rh^ (ocher) and C^Gr^ (purple) peak.

The differences in coverage between the CO adsorption (last data point in Figure 2a) and temperature‐programmed experiment (first data point in Figure 2b) are again assigned to beam‐induced dissociation. Furthermore, comparison of the coverage of the C 1s core level spectra recorded at 550 K of the as‐prepared and precovered Fe‐NCs, show that the ratio of Fe_3_C and Fe_3_C^surface^ differs significantly (Figure 4). The Fe_3_C^surface^ signal corresponded to 0.013 ML for the as‐prepared clusters at 550 K, while for the precovered Fe‐NCs it decreased to 0.006 ML. The reason why less surface carbide is observed is assumed to be due to restructuring of the Fe‐NCs resulting in a smaller surface area. We rule out possible sintering of the Fe‐NCs because the amount of empty pores in the N 1s spectrum after heating to 550 K is still 55±5 % (Figure S5). In case of sintering, an increase of the number of empty pores would be expected.^[27]^ Furthermore, the presence of carbon on iron clusters is known to reduce the mobility of the clusters and inhibit sintering.^[47]^ Instead, the restructuring of the Fe‐NCs is also supported by the peak shape in the spectra shown in Figure 4. The Fe_3_C peak of the as‐prepared Fe‐NCs is broad and it seems to consist of more than just the two Fe_3_C signals (Figure 4a). However, the spectrum of the precovered Fe‐NCs at 550 K clearly shows the main Fe_3_C signal and the shoulder of the surface Fe_3_C signal at the lower binding energy site, suggesting a restructuring to structural more similar nanoclusters (Figure 4b). For Pd nanoclusters on graphene/Rh(111) the decrease of the Pd 3d_5/2_ signal was reported as a result of restructuring.^[21]^ Similar changes in the intensity are also observed in the Fe 3p spectra (Figure S4), supporting a restructuring of the as‐prepared Fe‐NCs. Additionally, we cannot rule out that the formation of larger iron carbide regions in the clusters contributes to the sharper peak shape of the Fe_3_C peak and the smaller Fe_3_C^surface^ signal.

The desorption temperature of CO from the precovered NCs was estimated to be 280 K (Figure S2), which is 33 K lower than for the as‐prepared NCs. This is attributed to a change in the adsorption energy of CO because of the presence of Fe_3_C and iron oxides. The oxygen signal of the Fe‐NCs is attributed to iron oxides (Fe_x_O_y_) as the Fe 3p spectra reveal the feature at >53 eV, which is commonly observed for iron oxides (Figure S4).[^48^, ^49^, ^50^] We exclude the formation of homogeneous Fe_3_C clusters because the ratio of Fe (1.4 ML) and carbide (0.03 ML, Fe_3_C+Fe_3_C^surface^) is much larger than the 3 : 1 ratio of Fe_3_C. From the quantitative analysis, we find a CO^top^ coverage (0.045 ML, Figure 2d), which is larger than the number of Fe surface sites on Fe_3_C (3×0.013 ML, Figure 2b), which rules out that CO is solely adsorbed on iron carbide, in particular in view of the van der Waals diameter of CO, which is significantly larger than the Fe next neighbor distance.^[7]^

Furthermore, the desorption temperatures of CO in both TPXPS experiments is lower than the desorption temperature of CO on the iron single‐crystal surfaces Fe(100)[^2^, ^45^] and Fe(110),^[51]^ which is reported to be ∼400 K (at heating rates of 10 K/s[^2^, ^45^] and 12.5 K/s^[51]^). The difference to the literature value of the desorption temperature is assumed to be due to the different structure of the nanoclusters in comparison to single‐crystal surfaces. For the precovered Fe‐NCs, the desorption temperature of 280 K is similar to the temperature reported for Fe clusters on graphite of 250–300 K.^[19]^ Oh et al.^[19]^ also did not observe dissociation of CO and the low desorption temperature was explained by interactions of iron with the graphite substrate.

As already mentioned above, no CO dissociation is observed in the TPXPS spectra of the precovered Fe‐NCs, as concluded from the identical total carbon coverage of 0.05 ML before and after CO adsorption plus heating (Figure 2c and 2d). The reason why no dissociation occurs, is the blocking of the hollow/edge adsorption sites, which are the most active sites on the as‐prepared NCs.[^12^, ^20^, ^45^] UPS measurements and also computations of CO adsorbed on Fe(100) showed that not only the carbon atom but also the oxygen atom interacts with the iron when CO is adsorbed at the four‐fold hollow site in a lying‐down orientation, which leads to a weakened C−O bond.[^36^, ^45^] This suggests that only CO at hollow/edge sites is sufficiently reactive to dissociate.

**Figure 5:**
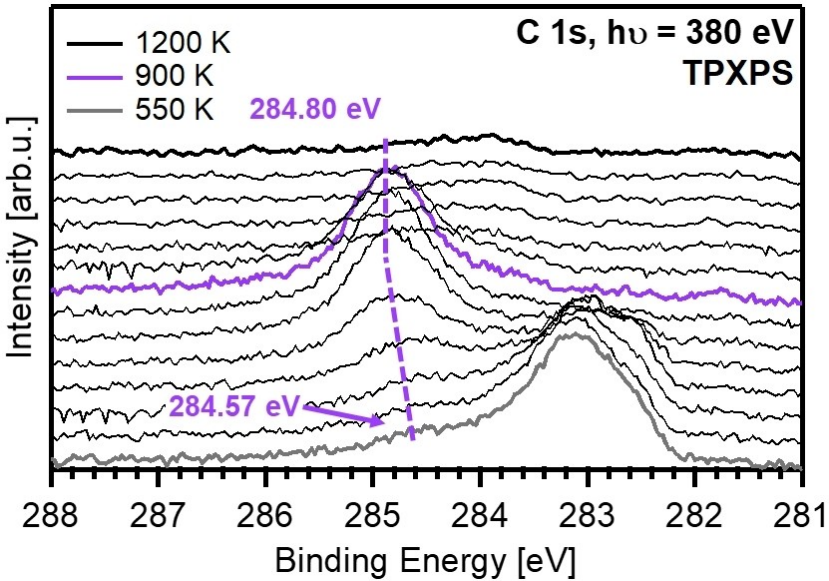
C 1s spectra of the TPXPS of the C‐ and O‐precovered Fe nanoclusters from 550–1200 K. The dashed lines indicate the shift of C^Gr^ (purple).

**Figure 6:**
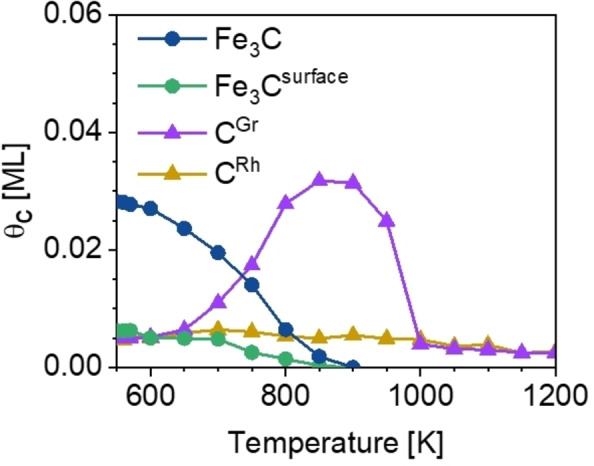
Quantitative analysis of the C 1s TPXPS of the C‐ and O‐precovered Fe nanoclusters in the temperature range of 550–1200 K (θ_C, max_=0.045 ML).

Finally, the sample was heated stepwise up to 1200 K; the corresponding C 1s spectra are depicted in Figure 5. The Fe_3_C present at 550 K reacts to form a new species. At 900 K, the main feature at 284.8 eV is assigned to graphitic carbon on iron (C^Gr^, purple); its C 1s binding energy is by 1.93 and 1.46 eV higher than that of Fe_3_C^surface^ and Fe_3_C, respectively.[^3^, ^52^, ^53^] C^Gr^ shifts from 284.57 to 284.80 eV with increasing temperature, while the signals of the iron carbides vanish (Figure 5). The formation of graphitic carbon on iron foils and powders is observed during carburization with synthesis gas for several hours at ∼520–550 K.^[50]^ For the Fe/*h‐*BN/Rh(111) system, C^Gr^ forms at around 700 K out of Fe_3_C (Figure 6, purple). One might also speculate whether C^Gr^ is due to a single graphite layer, that is, a graphene layer covering the Fe‐NCs. Although Vinogradov et al.^[54]^ reported that the formation of graphene out of iron carbide by annealing does not occur on Fe(110), the situation might be different for nanoclusters. The C 1s binding energy of C^Gr^ fits very well to the value of 284.9 eV for graphene on Fe(110).[^54^, ^55^] The encapsulation of Pt nanocluster arrays on *h‐*BN/Rh(111) with graphene was already reported by Düll et al.^[56]^ using ethylene as a precursor.

The quantitative analysis in the temperature range of 550–1200 K shows, that both iron carbide signals start to decrease at 600 K, while C^Gr^ increases. At temperatures above 900 K, C^Gr^ is removed from the surface due to recombination of carbon and atomic oxygen and the desorption of C‐ and O‐containing molecules. The desorption of recombined CO from iron single‐crystal surfaces at around 800 K is commonly observed.[^1^, ^2^, ^51^] Additionally, computations found that the recombination of C and O on iron is energetically favorable.^[14]^ Furthermore, the fact that C^Gr^ decreases at T>900 K may also support the assumption of graphene formation, since decomposition of graphene on Fe(110) was also observed to start at ∼900 K.^[54]^

## Conclusion

In this work, the adsorption sites and reactivity of CO on Fe‐NCs on *h‐*BN/Rh(111) were determined using in situ HR‐XPS. It was found that CO adsorbs at on‐top sites and hollow/edge sites of the iron clusters. The CO features in the C 1s spectra shift to higher binding energy with increasing CO exposure due to increasing lateral interactions of the CO molecules. The temperature programmed experiments show that CO starts to dissociate at temperatures above 300 K. The desorption temperatures of CO on the as‐prepared Fe‐NCs was determined to be 313 K.

On the C‐ and O‐precovered Fe‐NCs no adsorption of CO at hollow/edge sites was observed, since these sites were blocked by carbon and oxygen atoms. Furthermore, no further CO dissociation was found in the second TPXPS. Thus, the hollow/edge sites of the clusters were identified to be the most active sites for CO dissociation. We thus conclude that the catalytic activity of Fe‐NCs is inhibited by the C and O coverage. The desorption temperature of CO from the precovered Fe‐NCs was found to be 280 K, which is 33 K lower than for the as‐prepared Fe‐NCs. The change of the desorption temperature is a result of a change in the adsorption energy of CO on the precovered surface. Heating to temperatures above 650 K leads to the formation of graphitic carbon on iron. C^Gr^ is removed by increasing the temperature to 1000 K and the desorption of carbon and oxygen containing molecules.

## Conflict of interest

The authors declare no conflict of interest.

## Supporting information

As a service to our authors and readers, this journal provides supporting information supplied by the authors. Such materials are peer reviewed and may be re‐organized for online delivery, but are not copy‐edited or typeset. Technical support issues arising from supporting information (other than missing files) should be addressed to the authors.

Supporting Information
