# Supplementary material for: Reactivity and Passivation of Fe Nanoclusters on h‐BN/Rh(111)
Source: Chemistry. 2021 Sep 2;27(68):17087–93. doi: 10.1002/chem.202102590 (PMC9290904; doi:10.1002/chem.202102590)
Supplement: Supplementary file 1 — Supporting Information [file CHEM-27-17087-s001.pdf]

Chemistry–A European Journal

Supporting Information

Reactivity and Passivation of Fe Nanoclusters on *h*-BN/Rh(111)

Natalie J. Waleska, Fabian Düll, Philipp Bachmann, Felix Hemauer, Johann Steinhauer, and Christian Papp*

Parameters of the fitted XP spectra

CO Adsorption (as-prepared Fe-NCs):

Species	Binding Energy [eV]	Gaussian Width [eV]	Lorentzian Width [eV]	Asymmetry Factor
CO^{top}	285.34 / 285.77	0.66	0.19	0.19
$CO^{hollow/edge}$	284.41 / 284.92	0.66	0.19	0.19
C^{Gr}	284.57	0.66	0.13	0.11
C^{Rh}	283.81	0.66	0.13	0.11
Fe_3C	283.15	0.60	0.11	0.09
$Fe_3C^{surface}$	-	-	-	-

TPXPS (as-prepared Fe-NCs):

Species	Binding Energy [eV]	Gaussian Width [eV]	Lorentzian Width [eV]	Asymmetry Factor
CO^{top}	285.76 / 285.49	0.66	0.19	0.19
$CO^{hollow/edge}$	284.93 / 284.82	0.66	0.19	0.19
C^{Gr}	284.57	0.66	0.13	0.11
C^{Rh}	283.81	0.66	0.13	0.11
Fe_3C	283.17	0.60	0.11	0.09
$Fe_3C^{surface}$	282.82	0.46	0.06	0.01

CO Adsorption (precovered Fe-NCs):

Species	Binding Energy [eV]	Gaussian Width [eV]	Lorentzian Width [eV]	Asymmetry Factor
CO^{top}	285.62 / 285.73	0.66	0.19	0.19
$CO^{hollow/edge}$	-	-	-	-
C^{Gr}	284.57	0.66	0.13	0.11
C^{Rh}	283.81	0.66	0.13	0.11
Fe_3C	283.11	0.60	0.11	0.09
$Fe_3C^{surface}$	282.63	0.46	0.06	0.01

TPXPS (precovered Fe-NCs):

Species	Binding Energy [eV]	Gaussian Width [eV]	Lorentzian Width [eV]	Asymmetry Factor
CO^{top}	285.72 / 285.59	0.66	0.19	0.19
$CO^{hollow/edge}$	-	-	-	-
C^{Gr}	284.57 / 284.79	0.66	0.13	0.11
C^{Rh}	283.81	0.66	0.13	0.11
Fe_3C	283.16 / 282.98	0.66	0.11	0.09
$Fe_3C^{surface}$	282.73 / 282.56	0.46	0.06	0.01

N 1s spectra:

Species	Binding Energy [eV]	Gaussian Width [eV]	Lorentzian Width [eV]	Asymmetry Factor
Empty Pores	398.84	0.64	0.15	0.05
Filled Pores	398.60 / 398.45	0.68	0.03	0.09
Wires	398.15 / 398.00	0.65	0.08	0.04

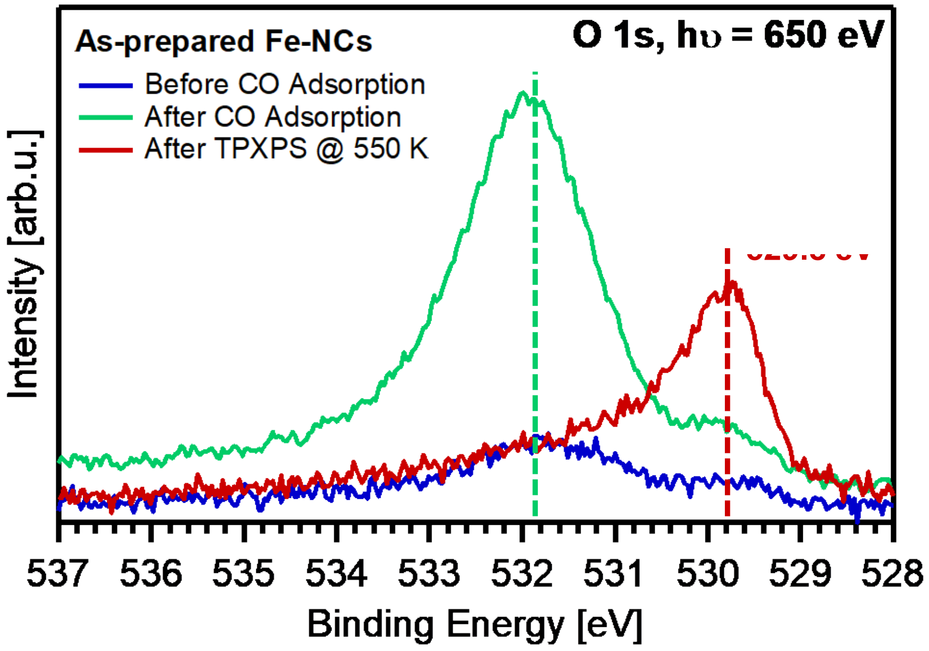

Figure S1: O 1s spectra of the CO adsorption and TPXPS of the as-prepared Fe-NCs. ($\theta_{O,\text{ before Ads.}}$ = 0.06 ML, $\theta_{O,\text{ after Ads.}}$ = 0.21 ML, $\theta_{O,\text{ 550 K}}$ = 0.10 ML). The signals at 531.9 and 529.8 eV are assigned to molecular CO and atomic oxygen, respectively.

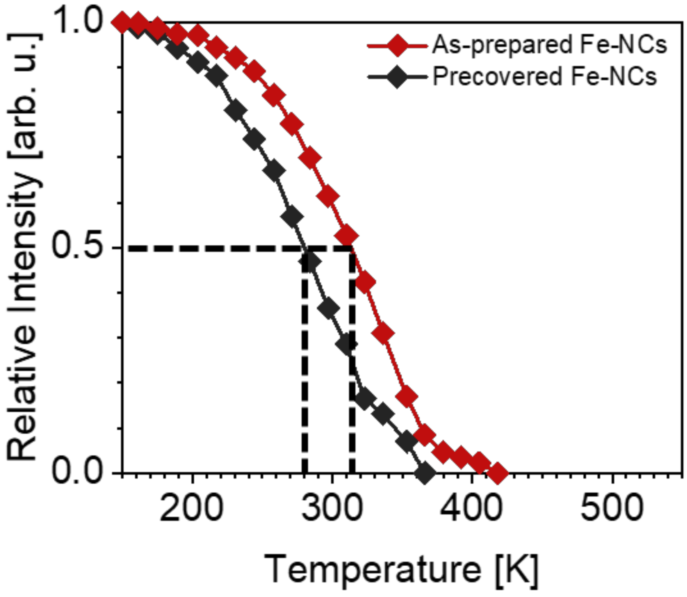

Figure S2: Determination of desorption temperature of the TPXPS. The desorption temperature of CO of the as-prepared Fe-NCs is 313 ± 5 K and 280 ± 5 K for the precovered Fe-NCs, as determined from the inflection point of the relative intensity of the CO signal, which corresponds to the rate maximum.

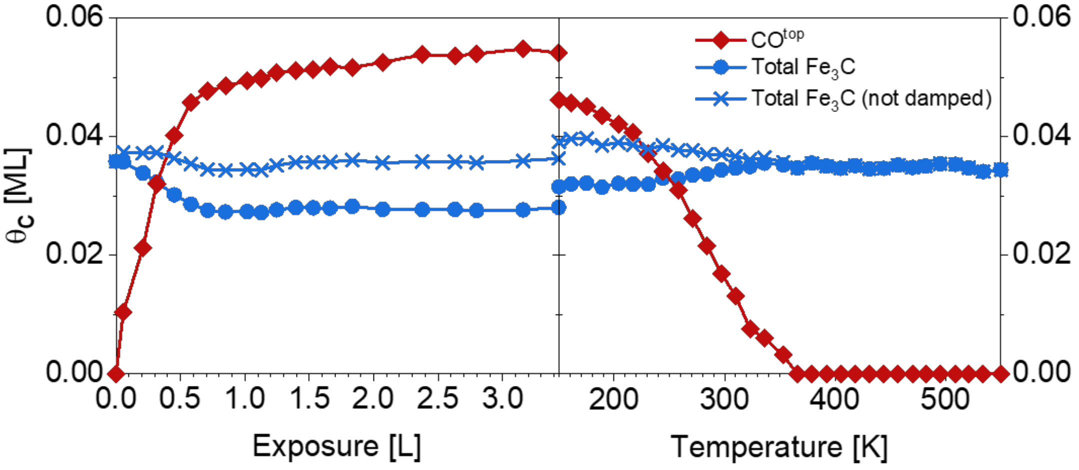

Figure S3: Determination of the damping of the Fe_3C signals by the adsorbed CO. The calculation gives an estimation of the undamped total Fe_3C coverage, showing that no loss of the carbide takes place during CO adsorption and TPXPS.

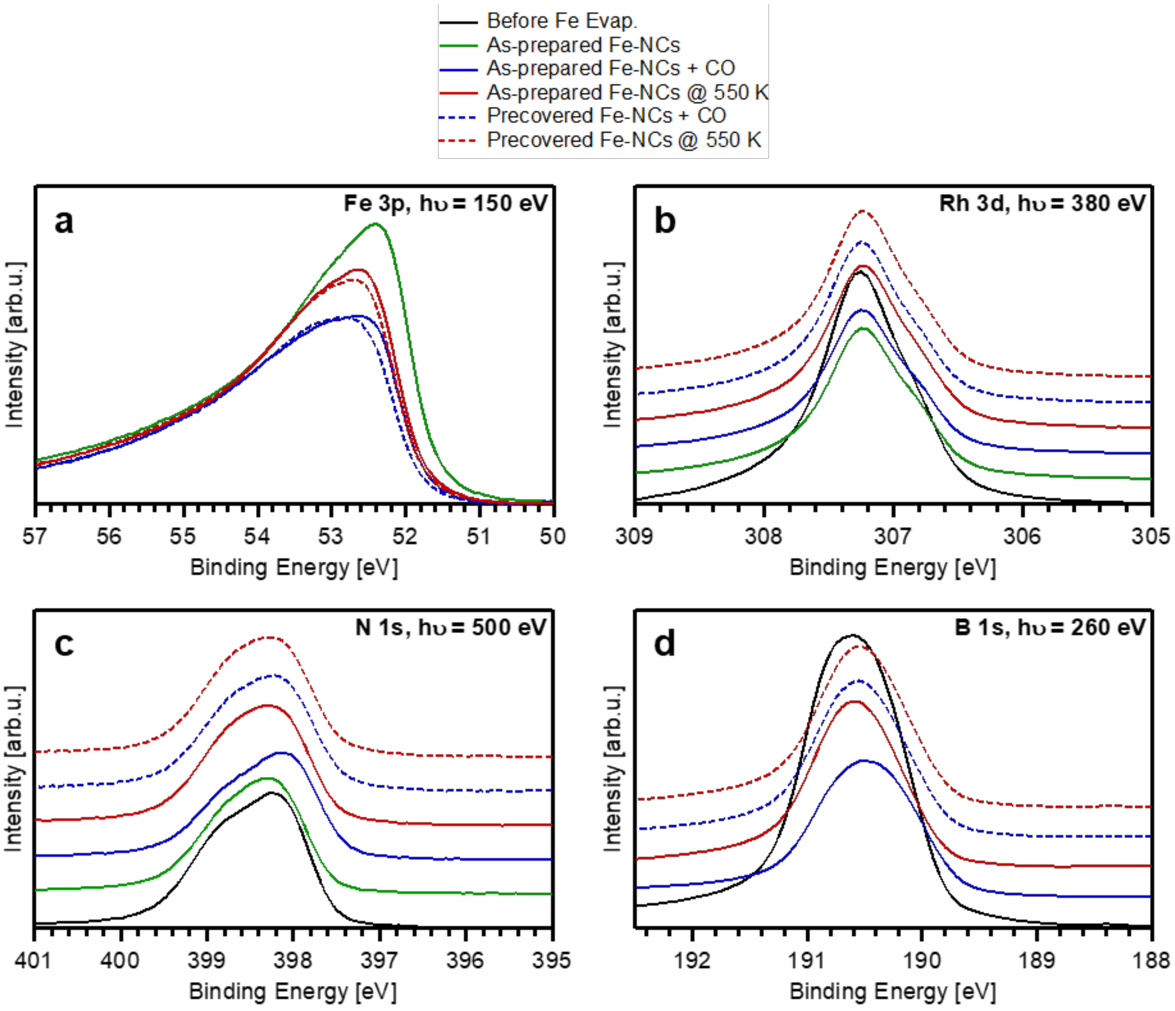

Figure S4: (a) Fe 3p, (b) Rh 3d, (c) N 1s and (d) B 1s spectra of the as-prepared Fe-NCs (full lines) and the precovered Fe-NCs (dashed lines). The blue spectra were recorded after CO adsorption and the red spectra after the TPXPS at 550 K.

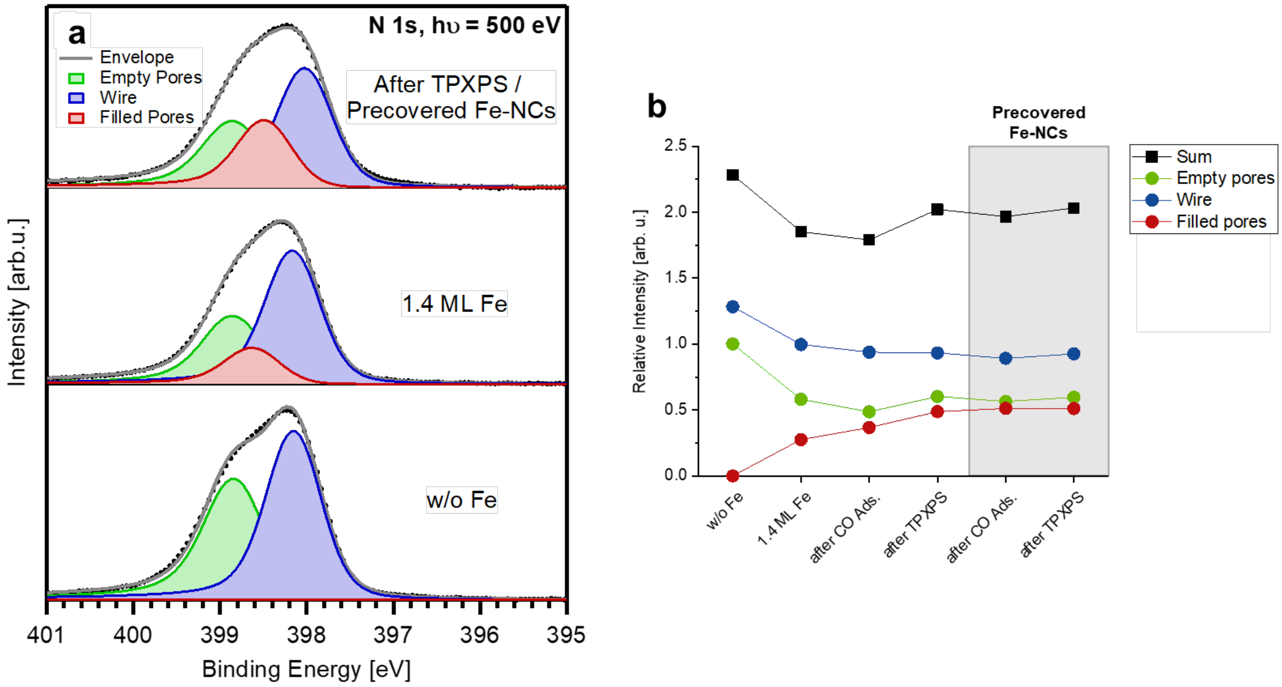

Figure S5: a) Selected spectra of the N 1s core level region including the fits of the spectra. b) Quantitative analysis of the N 1s spectra according to Düll *et al.*[1] After Fe deposition 55 ± 5 % of the empty pores remained. Thus, a number of ~370 Fe atoms per Moiré unit cell was determined. The spectra of the N 1s core level were used for the determination of the cluster size because the resolution of the peaks of the pores and wires is much better than for the B 1s spectra.

---

[1] F. Düll, M. Meusel, F. Späth, S. Schötz, U. Bauer, P. Bachmann, J. Steinhauer, H. P. Steinrück, A. Bayer, C. Papp, *Phys. Chem. Chem. Phys.* **2019**, *21*, 21287–21295.

SEM/XRF analysis of 1.4 ML Fe/*h*-BN/Rh(111)

To exclude the presence of clusters larger than the expected size of ~3 nm, a microscopic analysis of the sample was conducted, as well as an elemental analysis by XRF. The sample was prepared as described in the experimental section of the manuscript. However, a laboratory X-ray source was used (Al K_{α}, hv = 1486.6 eV) instead of synchrotron radiation.

The spectra shown in Figure S6 were recorded before and after the SEM experiments. In Figure S6a the spectra clearly show the presence of the Fe $2p_{1/2}$ and Fe $2p_{3/2}$ peaks at 720 and 707 eV. The difference of the spectra recorded before and after the SEM experiments is due to oxidation of the sample because it was transferred to the SEM apparatus exposed to the air. Thus, the green spectrum is lower in intensity due to damping of the carbon and oxygen present on the sample. Additionally, the Fe 2p spectrum is shifted to higher binding energy as a result of the oxidation of iron. Nevertheless, as the Fe $2p_{1/2}$ and Fe $2p_{3/2}$ peaks are still observable in the spectra after performing SEM, we can exclude the agglomeration of Fe-NCs to larger clusters. In case of agglomeration to a single particle, this single particle would have to have a diameter of ~60 µm, if it is assumed to be spherical. It would be possible to observe such particle using SEM. However, if this single particle would exist, no signal in the Fe 2p region would be observed because the surface of the particle would be too small in comparison to the overall investigated surface to obtain a signal in the corresponding core level region.

Furthermore, damping of the signals observed in the XP spectra after the SEM experiments is consistent in all core level regions (Figure S6b, c and d), while the presence of carbon and oxygen on the sample can be confirmed by the spectra of the C 1s and O 1s core level regions depicted in Figure S6e and f.

We investigated various regions on the sample microscopically. Exemplarily, Figure S7 shows SEM images of the sample at different magnifications and the corresponding elemental analysis by XRF is presented in Figure S8. In Figure S7a, a hexagonal shaped particle is shown with a diameter of ~2.3 µm, which was used to adjust the focus to get an image as clear as possible. After the adjustment of the focus, the sample was shifted to picture a homogenous area (Figure S8b). This was done because the Fe-NCs are expected to be spread homogenously over the surface, otherwise no Fe 2p peak would have been observed in the XP spectra. However, the resolution of the SEM was not good enough to image the ~3 nm small Fe-NCs. Figure S7c and d show the same area on the sample as depicted in Figure S7a and b with a lower magnification.

The frames added in Figure S7c and d mark the regions, where the images shown in Figure S7a (blue), b (red) and c (green) were recorded. Figure S7d shows that particles (bright spots) are present all over the surface. However, these defects only account for less than 1 % of the surface and thus have no influence on the XPS data and its analysis.

It was confirmed by the XRF analysis, that the bright spots observed in the SEM images are defects of the Rh surface. Figure S8 shows the recorded map at the same position as the SEM image was taken in Figure S7a. The analysis was performed for the elements Fe, Rh, N and B. Figure S8a shows another corresponding SEM image. In Figure S8b, d and e, the Fe, N and B regions no signal above the detection limit is found, while Figure S8c clearly shows that the particle fully consists of Rh. Please note, the dark spot on the right side of the defect is the shadow of the particle itself.

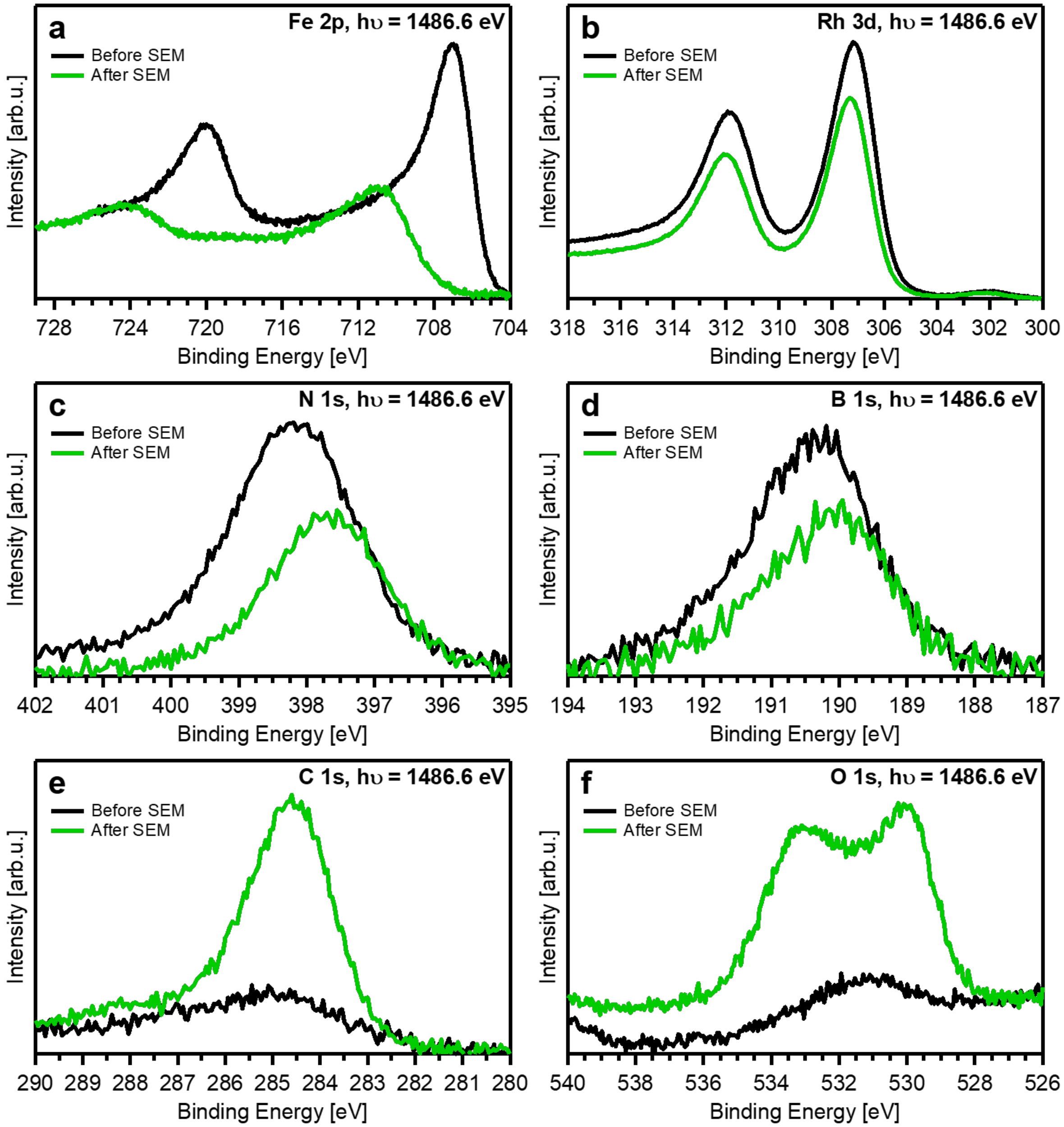

Figure S6: XP spectra of 1.4 ML Fe on the *h*-BN/Rh(111) substrate of the (a) Fe 2p, (b) Rh 3d, (c) N 1s, (d) B 1s, (e) C 1s and (f) O 1s core level regions. The spectra were recorded before (black) and after the SEM experiments (green).

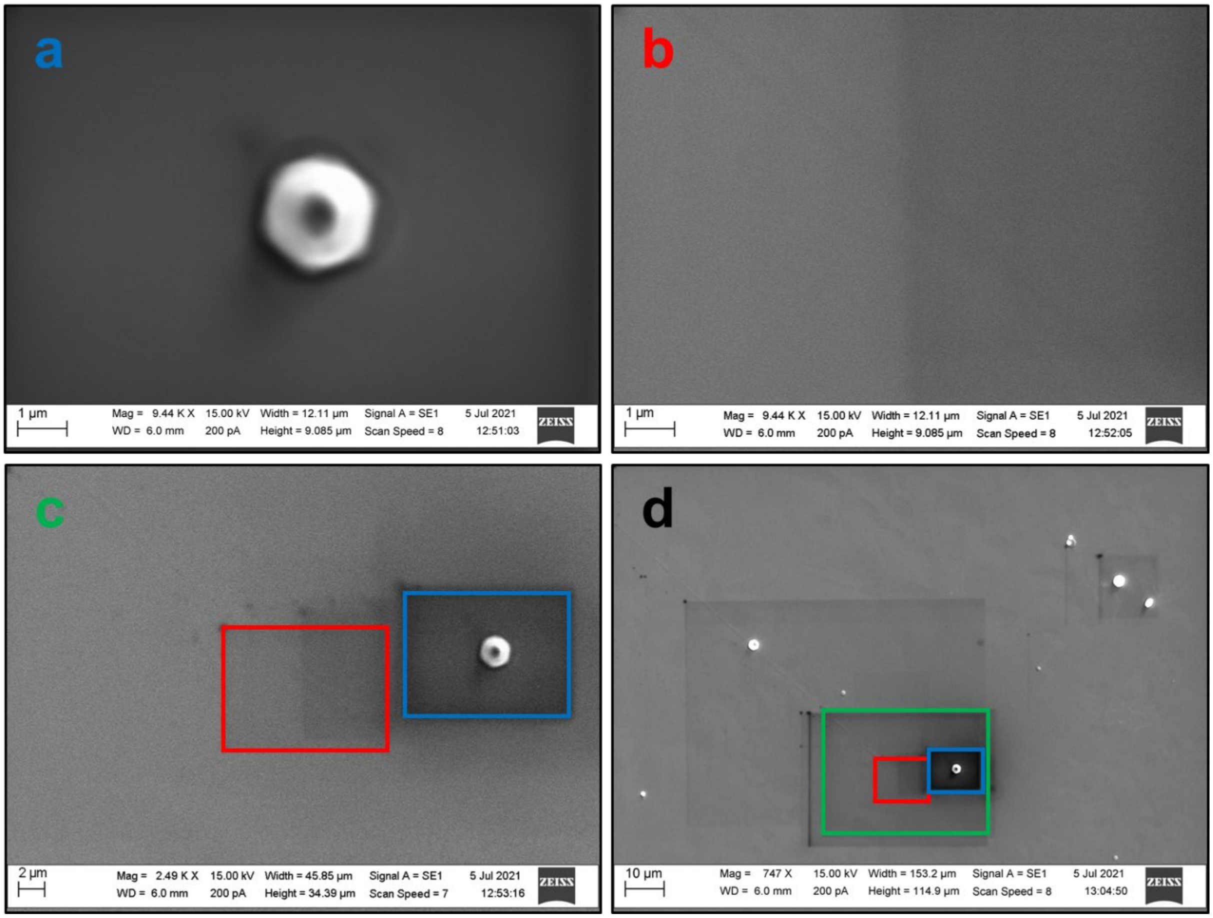

Figure S7: SEM images of the Fe/*h*-BN/Rh(111) recorded at different magnifications. For more information please refer to the text.

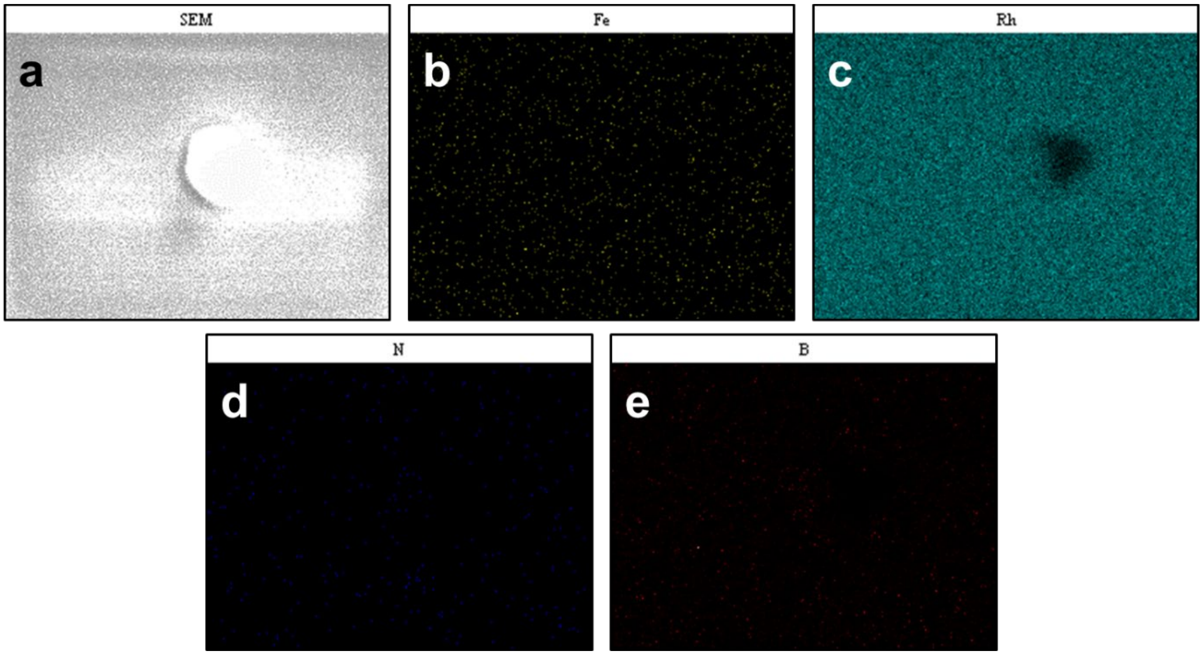

Figure S8: XRF analysis of the Fe/h-BN/Rh(111) sample. (a) The analyzed spot corresponds to the same area shown in Figure S7a. (b), (c), (d) and (e) show the elemental composition of the sample for Fe (yellow), Rh (turquoise), N (blue) and B (red), respectively.
